# MicroRNA-132 regulates quinolinic acid production in the brain during LPS-induced neuroinflammation

**DOI:** 10.3389/fimmu.2025.1644783

**Published:** 2025-08-26

**Authors:** Amir Mohamed Kezai, Papa Yaya Badiane, Benjamin Hennart, Delphine Allorge, Sabrina Marion, Sébastien S. Hébert

**Affiliations:** ^1^ Centre Hospitalier Universitaire (CHU) de Québec Research Center, Québec City, QC, Canada; ^2^ Department of Psychiatry and Neurosciences, Laval University, Québec City, QC, Canada; ^3^ Centre Hospitalier Universitaire (CHU) Lille, Unité Fonctionnelle de Toxicologie, Lille, France; ^4^ Lille University, Centre National de la Recherche Scientifique (CNRS), Inserm, Centre Hospitalier Universitaire (CHU) Lille, Institut Pasteur de Lille, U1019-UMR9017-CIIL- Center for Infection & Immunity of Lille, Lille, France

**Keywords:** microRNA-132, kynurenine pathway, kynurenine 3-monooxygenase, quinolinic acid, neuroinflammation

## Abstract

**Background:**

The kynurenine pathway (KP) plays a major role in neuroinflammation by converting the amino acid tryptophan into a variety of neuroactive products, including neurotoxic quinolinic acid (QUIN). The gene expression regulatory role of microRNAs in neuroinflammation is well documented; however, their impact on KP in the brain remains unexplored.

**Methods:**

In this study, we investigated whether the neuroimmune miR-132/212 cluster regulates one or more members of the KP during lipopolysaccharide (LPS)-induced neuroinflammation *in vivo* in mice and *in vitro* in BV-2 microglial cells.

**Results:**

In wildtype mice, we demonstrated that a subtoxic dose of LPS triggers a significant neuroinflammatory response with upregulation of KP enzymes, particularly kynurenine 3-monooxygenase (KMO), a key enzyme in QUIN synthesis, leading to elevated brain levels of this neurotoxic metabolite. Interestingly, KMO expression and activity remained elevated in miR-132/212 knockout mice after post-inflammation resolution. *In vitro* experiments using BV-2 microglial cells showed that miR-132 overexpression led to downregulation of KMO expression and enzyme activity and reduced QUIN levels without altering the microglial activation status.

**Conclusion:**

Collectively, these findings suggest that the miR-132/212 cluster functions as a novel modulator of KP metabolism during LPS-induced inflammation, and acts as a potential therapeutic target for controlling neurotoxic QUIN accumulation in neuroinflammatory conditions.

## Introduction

The kynurenine pathway (KP) plays a key role in central nervous system (CNS) inflammation. Within this pathway, tryptophan (TRP) is converted to kynurenine (KYN) by the enzymes indoleamine 2,3-dioxygenase 1 and 2 (IDO-1/2) and tryptophan 2,3-dioxygenase (TDO). In astrocytes, KYN is further metabolized into the neuroprotective compound, kynurenic acid (KYNA), by kynurenine aminotransferases (KATs). In microglia, on the other hand, KYN is processed into 3-hydroxykynurenine (3-HK) through the action of kynurenine 3-monooxygenase (KMO) (1). 3-HK is then converted into 3-hydroxyanthranilic acid (3-HAA) and subsequently into quinolinic acid (QUIN), a neurotoxic metabolite and potent N-methyl-D-aspartate (NMDA) receptor agonist (2). 3-HK can also form xanthurenic acid (XA), a structural analog of KYNA, through the action of KATs (3). Under normal conditions, brain KYN metabolism tends to favor KYNA production. However, during homeostatic disturbances, this balance can shift toward increased QUIN production. Owing to its ability to enhance excitatory glutamatergic signaling, prolonged elevated QUIN levels can result in neuronal death, tissue damage, and seizures (4). Notably, certain brain regions are more vulnerable to QUIN-induced excitotoxicity (5).

Neuroinflammation is a key activator of KP (6–10). A well-established method for inducing neuroinflammation and KP activation is lipopolysaccharide (LPS) treatment. LPS treatment has been previously shown to induce the expression of several key enzymes of the KP pathway, including indoleamine 2,3-dioxygenase-1 (IDO-1), KMO, kynureninase (KYNU), and 3-hydroxyanthranilate 3,4-dioxygenase (HAAO) in different brain regions and murine microglial cell cultures (7, 11–13). Furthermore, LPS treatment induced an increase in QUIN levels in different brain regions in mice (8, 14).

The small (21–25 nucleotides) noncoding and evolutionarily conserved microRNAs (miRNAs) are critical mediators of neuroinflammation. They act as fine tuners by either promoting or inhibiting inflammatory processes in different cell populations, including neurons, microglia, and astrocytes (15–21). The miR-132/212 cluster is of particular interest because of its neuroimmune functions [reviewed in (22)]. Notably, miR-132 acts as an anti-inflammatory regulator in the brain by suppressing inflammation through acetylcholinesterase gene targeting (23, 24). In the context of Alzheimer’s disease (AD), a neurodegenerative disease associated with chronic neuroinflammation, the miR-132/212 cluster is strongly downregulated in the human brain, whereas miR-132 overexpression in AD mouse and cellular models protects against disease pathologies and cognitive loss through multiple signaling pathways (25–28). MiR-212 upregulation reduces neuroinflammation and suppresses pyroptosis of neurons by regulating the activation of the nucleotide-binding oligomerization domain-like receptor family pyrin domain containing 3 (NLRP3)/Caspase-1 signaling pathway (29). Interestingly, miR-132 was shown to attenuate LPS-induced inflammation in alveolar macrophages by targeting the nuclear factor kappa-light-chain-enhancer of activated B cells (NF-κB) and Janus kinase 2 signal transducer and activator of transcription 3 (JAK2/STAT3) signaling (30). Moreover, miR-132 inhibits LPS-induced pro-inflammatory cytokine production by targeting tumor necrosis factor receptor-associated factor 6 (TRAF6), transforming growth factor-activated protein kinase 1 (TAK1), and TAK1 binding protein 1 (TAB1) in Teleost Fish (31). MiR-132 and miR-212 expression is upregulated by LPS in patients with Hirschsprung’s disease patients inducing Sirtuin 1 suppressing and NLRP3 inflammasome activation (32). Finally, miR-212 suppresses LPS-induced inflammatory responses in macrophage RAW264.7 cells by targeting the HMGB1 gene (33).

The potential link between the KP and its modulation by miRNAs remains poorly explored. One previous study showed that miR-126-5p directly targets tryptophan 2,3-dioxygenase (TDO2), a highly expressed KP enzyme in the liver, and promotes intracellular tryptophan metabolism (34). Additionally, miR-874-3p prevented LPS-induced depression-like behavior in mice by inhibiting IDO-1 expression (35). To date, the role of miRNAs in targeting other key enzymes of the KP pathway remains unknown, especially in the brain. In this study, we explored the possibility that the neuroimmune miR-132/212 cluster regulates one or more members of the KP following LPS-induced neuroinflammation. Our results showed that *in vivo* in mice, LPS triggered robust neuroinflammation and KP activation, with elevated QUIN levels. miR-132/212 knockout (KO) mice failed to normalize KMO and KATII expression, and activity post-LPS treatment. MiR-132 directly suppressed KMO expression in cultured microglia, thereby reducing QUIN production. Collectively, these findings reveal miR-132 as a potent new, stress-mediated modulator of the KP by targeting KMO.

## Materials and methods

### Animals

miR-132/212 KO mice, a kind gift from Dr. Richard H. Goodman (Oregon Health & Science University, USA), were generated as previously described (36). Male miR-132/212 KO mice and wild-type littermate controls of the same sex at 6 months of age (n= 19-20/genotype) were injected with 200 μl of LPS (from Escherichia coli O111:B4: L5293; Sigma, Canada) at a concentration of 750 μg/kg and 0.9% sterile sodium chloride (Teligent, Canada). Mice were sacrificed without anesthesia after 6 and 24 hours post-LPS-treatment, and the brains were removed from the skull and dissected on ice. Tissues were frozen on dry ice and stored at −80°C until use. All animal procedures were conducted according to the Canadian Council on Animal Care (CCAC) guidelines, as administered by le Comité de Protection des Animaux de l’Université Laval CPAUL 3 (#2023-1304, CHU-23-1304).

### MicroRNA quantification

Total RNA was extracted from mouse brains using TRIzol Reagent (Life Technologies) according to the manufacturer’s instructions. TaqMan^®^ miRNA assay (Life Technologies) was used for miR-132 (#000457) and miR-212 (#000515) quantification, following the manufacturer’s protocol. RNU48 (#001006) or RNU19 (#000391), as indicated in the text, were used as normalization controls. Relative expression was calculated using the 2^−ΔΔCT^ method.

### Real-time quantitative PCR analysis

The left hemispheres of mouse brains were sacrificed and frozen at -80°C after 6 and 24 h post-LPS treatment and were powdered on dry ice to prevent thawing. The powder (80 mg) was used to extract RNA using TRIzol Reagent (Life Technologies) following the manufacturer’s recommendations. RNA concentration and quality were measured using a NanoQuant plate (Infinite 2000, TECAN) spectrophotometer. Two micrograms of RNA was used to synthesize cDNA by reverse transcription using the High-Capacity cDNA Reverse Transcription Kit (ThermoFisher, 4368814) according to the manufacturer’s protocol. Thirty nanograms of cDNA was used for each RT-qPCR reaction, containing the sense and antisense primers of the genes of interest (**Table 1**) at a final concentration of 1 µM. Primer’s specificity is verified by sequence alignment on NCBI Primer-BLAST (https://www.ncbi.nlm.nih.gov/tools/primer-blast/). The RT-Qpcr reaction was carried out in the presence of PowerTrack SYBR Green Master Mix (ThermoFisher) using the LightCycler 480 II (Roche) with the following PCR program: 50°C 2 min, 95°C 10 min, [95°C 30sec, 60°C 30sec] x40, 95°C 1 min, 55°C 30sec, 95°C 30sec. Relative expression was calculated by the 2−^ΔΔCT^ method.

**Table 1:** Primers used for RT-qPCR.

Gene	Forward	Reverse
GAPDH	ACAAAATGGTGAAGGTCGGTG	TGGCAACAATCTCCACTTTGC
IL-12	ACCAAATTACTCCGGACGGT	TGGTCCAGTGTGACCTTCTC
TNFα	TGCCTATGTCTCAGCCTCTTC	GAGGCCATTTGGGAACTTCT
IFNγ	CAGCAACAGCAAGGCGAAA	TTCCTGAGGCTGGATTCCG
IDO-1	GTAGAGCGTCAAGACCTGAAAG	GATATATGCGGAGAACGTGGAA
KATII	GTTCTCCACACACAAGTCTCA	AAACCCTCTTCTCCCCATTG
KMO	TTCCACCTGAAGTCACACTG	ACCAAGCAGTCTTCAAAGCC

### Proteomic QUIN pathway analysis

Once removed, a half-brain was placed in a sterile bead tube (Precellys, Bertin Instruments) containing 1mL of cold sterile PBS 1X supplemented with perchloric acid 10% and crushed using the Retsch MM 400 mixer at 5000 rpm (2x 15 sec and 15sec pause). The lysate was centrifuged at 14,000 rpm for 10 min at 4°C, and the supernatant was stored at -80°C until the metabolites were assayed by the toxicology department at Lille University Hospital, France. Briefly, the assay was performed using liquid chromatography coupled with tandem mass spectrometry (LC-MS/MS). Protein precipitation (50 μL test sample) was performed with 50 μL of acetonitrile containing tryptophan-D5 (50 μM, CDN isotopes, Pointe-Claire, Canada) used as an internal standard. After centrifugation (10 min, 14000 g), 50 μL of the supernatant was diluted in 600 μL of water before injection into a UPLC-MS/MS system (Acquity TQ-XS, Waters, Milford, MA) equipped with a C18-XB column (1.7μm 100 column A 150 x ×.1 mm - Phenomenex). The ions of each compound were analyzed in ESI+ mode and monitored for MRM transitions. Masslinks software (Waters) was used for data acquisition and processing. The obtained concentrations were normalized to the weight of each half-brain measured before grinding.

### Immunofluorescence

Mouse brains recovered 6 hours post-LPS treatment were coronally sectioned into two hemispheres. The right hemisphere was fixed in 4% paraformaldehyde (PFA) for 12 h and then transferred to PBS-sucrose 30% for 24 h for cryopreservation. The half-brains were then transferred to isopentane at -40°C on dry ice for 10 s for rapid freezing. The half-brains were kept at -80°C until cold sectioning with a cryostat microtome (thickness, 35 μm). The resulting cryosections were preserved from contamination in PBS-Azide 0.2% until immunostaining. Sections were permeabilized with PBS-Triton 0.2% and saturated with Normal Goat Serum 1/100 (Vector Laboratories; Cat. No. S-1000) for one hour at room temperature and then labeled with primary antibodies anti-Iba1 1/500 (Rabbit, 019-19741, Wako) and anti-QUIN 1/200 (mouse, IS002, 4E11G3, ImmuSmol). Afterwards, sections were incubated with secondary antibodies goat anti-rabbit 568 1/500 (A11036, Invitrogen) and goat anti-mouse 488 1/500 (A11029, Invitrogen) for 1 hour at RT. The sections were then incubated with DAPI (D1306, Invitrogen) and bathed in 70% ethanol and Sudan black (Sigma, Canada) to eliminate autofluorescence. The sections were then mounted on Superfrost slides (ThermoFisher) using Fluoromount-G™ Mounting Medium (Invitrogen, 00495802). The slides were analyzed using an LSM 800 microscope (Zeiss) and Zen 3.9 software (Zeiss).

### Cell culture

Murine BV-2 microglial cells were purchased from Cell Lines Service (Cytion, catalog number 305156). Upon receipt, BV-2 microglia were cultured in T25 flasks (Sarstedt) containing Roswell Park Memorial Institute (RPMI 1640) medium (Wisent Inc, Canada) supplemented with 10% fetal bovine serum and 1% penicillin–streptomycin (SH40003.01, Cytiva, Austria) at 37°C in a humidified atmosphere containing 5% CO2. After four passages, BV-2 microglia were used for transfection and immunostaining experiments. The human cell line HEK 293 was purchased from ATCC (CRL-1573) and cultured in T25 flasks (Sarstedt) containing Dulbecco’s Modified Eagle Medium (DMEM) (Wisent Inc, Canada) supplemented with 10% fetal bovine serum and 1% penicillin–streptomycin at 37°C in a humidified atmosphere containing 5% CO2. HEK 293 cells were used for the luciferase reporter assay.

### Microglia transfection

BV-2 microglia were seeded in 24-well flat-bottom plates (FB012929, Fisherbrand) containing RPMI 1640 supplemented with 10% FBS without antibiotics for 48 h at 37°C in a humid atmosphere containing 5% CO2. Seventy to 80% confluent cells were transfected with 50 nM pre-miR hsa-miR-132 (AM17100, Invitrogen) using Lipofectamine RNAiMAX (Invitrogen, 13778030). A scrambled miRNA mimic (AM17111, ThermoFisher) was used as a negative control. After viability checking, cells were processed for RT-qPCR, immunostaining, and Western Blotting 48 h post-transfection.

### Dual luciferase assay

A LUC-pair dual-luciferase assay was conducted to validate the binding of miR-132 to the KMO 3′ untranslated region (3’UTR). Briefly, HEK 293 cells were seeded in 24-well flat-bottom plates (FB012929, Fisherbrand) containing DMEM (Wisent Inc., Canada) supplemented with 10% FBS without antibiotics for 48 h at 37°C in a humid atmosphere containing 5% CO2. Seventy to 80% confluent cells were then transfected with 50 nM pre-miR hsa-miR132 (AM17100, Invitrogen) and 100 ng of miRNA 3’ UTR murine KMO luciferase victor (MmiT037508-MT06 pEZX-MT06-KMO, GeneCopoeia, USA) using Lipofectamine 2000 (11668-019, Invitrogen). A scrambled miRNA mimic (AM17111, ThermoFisher) and CmiT000001-MT06 victor (GeneCopoeia, USA) were used as negative controls. Cells were incubated for 48 h post-transfection before harvesting, and luciferase activities (Firefly and Renilla) were measured according to the manufacturer’s instructions (Luc-Pair Duo-Luciferase Assay Kit 2.0 LF001, GeneCopoeia, USA). Relative luciferase activity was calculated by normalizing it to Renilla luciferase activity.

### Western blotting

BV-2 microglia transfected with pre-miR-132 mimics (AM17100, Invitrogen) or scrambled mimics (AM17111, ThermoFisher) were lysed with RIPA buffer: 50 mM Tris–HCl (pH 8), NaCl/1 mM, nonidet P-40, sodium deoxycholate, SDS, phosphatase inhibitors 1 mM, PMSF 1mM and a Complete Mini EDTA-free Protease Inhibitor Cocktail tablet (Roche Life Science). The extracts were centrifuged at 13,000 rpm for 10 min. Protein concentrations in the supernatant were measured using the Pierce BCA Protein Assay Kit (ThermoFisher). Ten micrograms of proteins were resolved using 10% SDS-PAGE gels and transferred onto nitrocellulose membranes (Bio-Rad). Membranes were blocked with 5% non-fat dry milk in TBS 0.1%/Tween-20 for 1 h at room temperature, and then incubated overnight at 4°C with the primary antibodies anti-KMO 1/1000 (Rabbit, Proteinteck) and anti-GAPDH 1/5000 (MAB374, Sigma). Bound antibodies were revealed by enhanced chemiluminescence detection using an HRP-conjugated secondary antibody anti-mouse 1/5000 (115-035-146, Jackson ImmunoResearch) and anti-rabbit 1/5000 (111-035-144, Jackson ImmunoResearch). The ChemiDoc imaging system (Bio-Rad) was used for immunoblotting. Densitometry was performed to quantify the signal intensity using the ImageJ v1.53 software.

### BV-2 microglia immunostaining

Bv-2 microglia transfected with pre-miR-132 or scrambled were cultured on glass coverslips, fixed with 4% PFA for 20 min, and kept at 4°C in PBS 1X. After 48 h, PFA was inactivated with 50 mM PBS/NH4Cl, and the cells were permeabilized with 0.1% PBS-Triton and saturated with 1% PBS-BSA. BV-2 microglia were incubated with primary antibodies anti-QUIN 1/200 (mouse, IS002, 4E11G3, ImmuSmol) and anti-Iba1 + 1/200 (Rabbit, 019-19741, Wako) overnight at 4°C followed by incubation with secondary antibodies anti-rabbit 568 1/500 (A11036, Invitrogen), anti-mouse 488 1/500 (A11029, Invitrogen) and DAPI 1/5000 (D1306, Invitrogen). Coverslips were then mounted on slides using Fluoromount-G™ Mounting Medium (Invitrogen, 00495802). The slides were analyzed with the LSM 800 microscope (Zeiss) using Zen 3.9 software (Zeiss). The quantification of QUIN and Iba1+ staining in both conditions is expressed as corrected total cell fluorescence (CTCF) normalized to the non-treated condition using ImageJ 1.53 software.

### Statistics

Statistical significance of RT-qPCR, western blotting, and luminescence quantification was determined using one-way ANOVA with Tukey’s *post hoc* test or unpaired two-tailed Student’s t-tests, as indicated in the text. Calculations were performed using GraphPad Prism 9 software and statistical significance was set at p < 0.05.

## Results

### LPS stimulation induces miR-132/212-dependent KP activation and QUIN formation in the mouse brain

**Figure 1:**
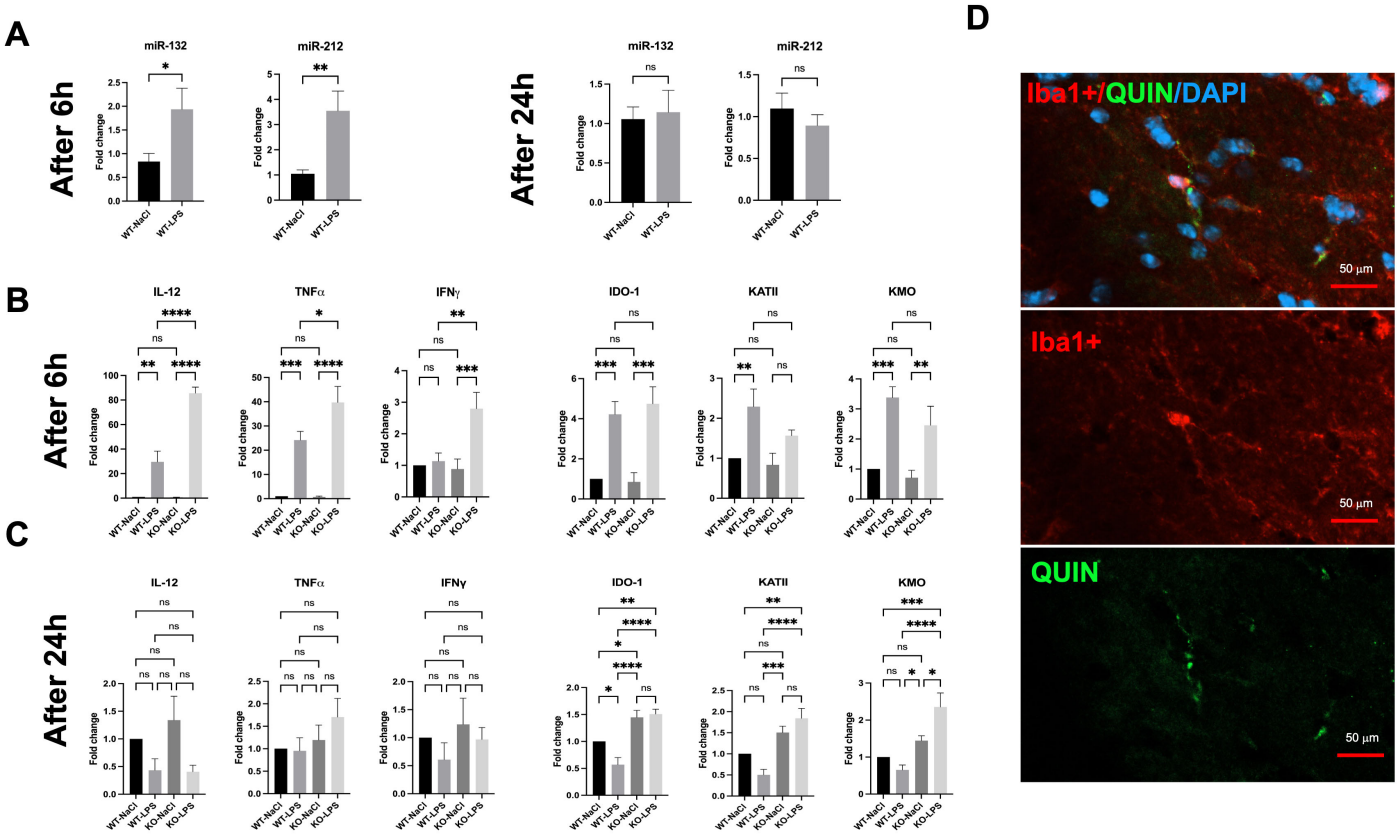
Effects of LPS on brains of WT and miR-132/212 knockout mice 6h and 24h post-stimulation. **(A)** RT-qPCR gene expression analysis of miR-132 and miR-212 in the brains of WT mice stimulated with LPS for 6h and 24h. The results are expressed as fold changes and compared with those of WT mice injected with NaCl. **(B)** RT-qPCR gene expression analysis of pro-inflammatory cytokines (IL-12, TNF-α, IFNγ) as well as kynurenine pathway enzymes (IDO-1, KMO, KATII) in the brains of WT and miR-132/212 KO mice stimulated with LPS for 6h and **(C)** 24h. The results are expressed as fold changes and compared with those of WT mice injected with NaCl. **(D)** Immunofluorescence results of the markers Iba1+ (red), QUIN (green), and DAPI (blue) on brain cryosections of miR-132/212 KO mice treated with LPS for 6h. All graphs show mean values ± SEMs (n= 19-20/genotype). The values were compared using one-way ANOVA. Asterisks (*) indicate significant differences. *p < .05, **p < .01, ***p < .001 and ****p < .0001. ns, not significant.

The *miR-132/212* cluster comprises two homologous miRNAs that are co-expressed from the same locus on chromosome 11 in mice (chromosome 17 in humans). To investigate the effects of LPS on miR-132 and miR-212 expression *in vivo*, we measured their levels in the brain of wildtype (WT) mice at 6 and 24h post-LPS treatment (**Figure 1A**). Our analyses revealed an increase in both miR-132 and miR-212 expression at 6h post-LPS treatment, followed by a return to baseline levels at 24h. This result is consistent with earlier findings (32, 37), indicating a transient effect of LPS stimulation on miR-132 and miR-212 expression.

Next, we investigated the *in vivo* effects of miR-132/212 deletion on LPS-mediated inflammation. We confirmed that LPS treatment induced a strong neuroinflammation 6h post-injection in WT mice, characterized by a 20-to-30-fold increase in the mRNA levels of pro-inflammatory cytokines IL-12 and TNF-α (**Figure 1B**). Interestingly, IL-12 and TNF-α expression reached significantly higher levels in miR-132/212 knockout (KO) mice after LPS treatment, up to 40- and 80-fold, respectively. Notably, IFNγ expression was induced by LPS treatment only in miR-132/212 KO mice.

As anticipated, LPS-induced neuroinflammation induced the expression of key enzymes of the KP in WT mice, including IDO-1, KMO, and KATII, with fold changes ranging from 1.5 to 4.5 (**Figure 1B**). Correlated with the up-regulation of the KP genes, at 6h post-treatment with LPS, QUIN production by microglia was observed *in vivo* by immunohistochemistry on brain sections from KO mice (**Figure 1D**). No significant differences in the basal expression of these KP genes were observed between WT and miR-132/212 KO mice. Similar results were obtained following LPS injection (**Figure 1B**). By 24h post-LPS treatment (**Figure 1C**), the expression levels of IL-12, TNF-α, and IFNγ returned to baseline levels in both WT and KO mice. Surprisingly, however, IDO-1 and KMO expression, and to a lesser extent KATII expression, remained elevated under miR-132/212 KO conditions at 24h post treatment.

**Figure 2:**
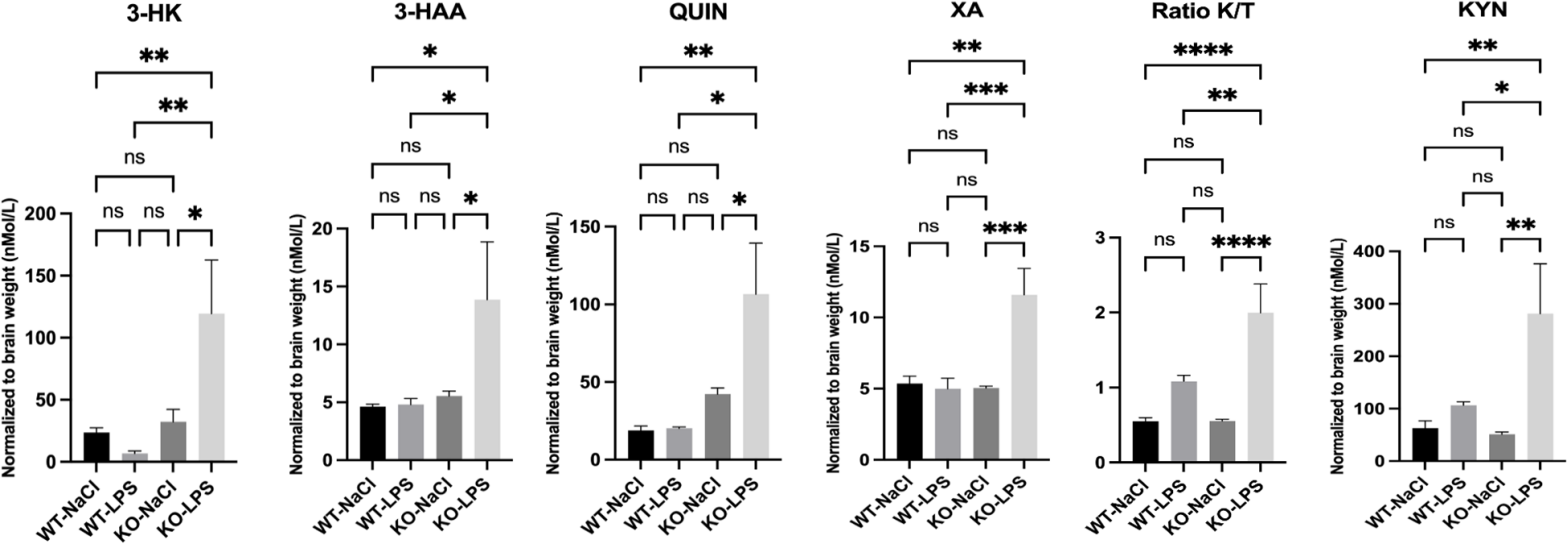
LC-MS/MS assay of kynurenine pathway metabolites (3-HK, 3-HAA, QUIN, XA KYN/TRP (K/T) ratio, and KYN) in the brains of WT and miR-132/212 knockout (KO) mice stimulated with LPS for 24h. All graphs show mean values ± SEMs. The values were compared using one-way analysis of variance (ANOVA). Asterisks (*) indicate significant differences. *p < .05, **p < .01, and ****p < .0001. ns, not significant.

To further assess the functional impact of miR-132/212 deficiency *in vivo*, we quantified KP metabolites in the brain of mice in basal condition and following 24h of LPS stimulation. The levels of metabolites resulting from KMO activity, including 3-HK, 3-HAA, and QUIN, were elevated in miR-132/212 KO mice (**Figure 2**). Increased KATII expression correlated with higher levels of the metabolite XA but not KYNA (not shown). Finally, the KYN/TRP (K/T) ratio also increased in miR-132/212 KO mice compared to WT mice. These results indicate a strong degradation of TRP to KYN only in miR-132/212 KO mice after 24h of LPS stimulation. Collectively, these findings demonstrate that the miR-132/212 cluster is essential for the proper regulation of KP activity in the brain *in vivo*.

### miR-132 modulates KMO expression and enzyme production in BV-2 microglia

**Figure 3:**
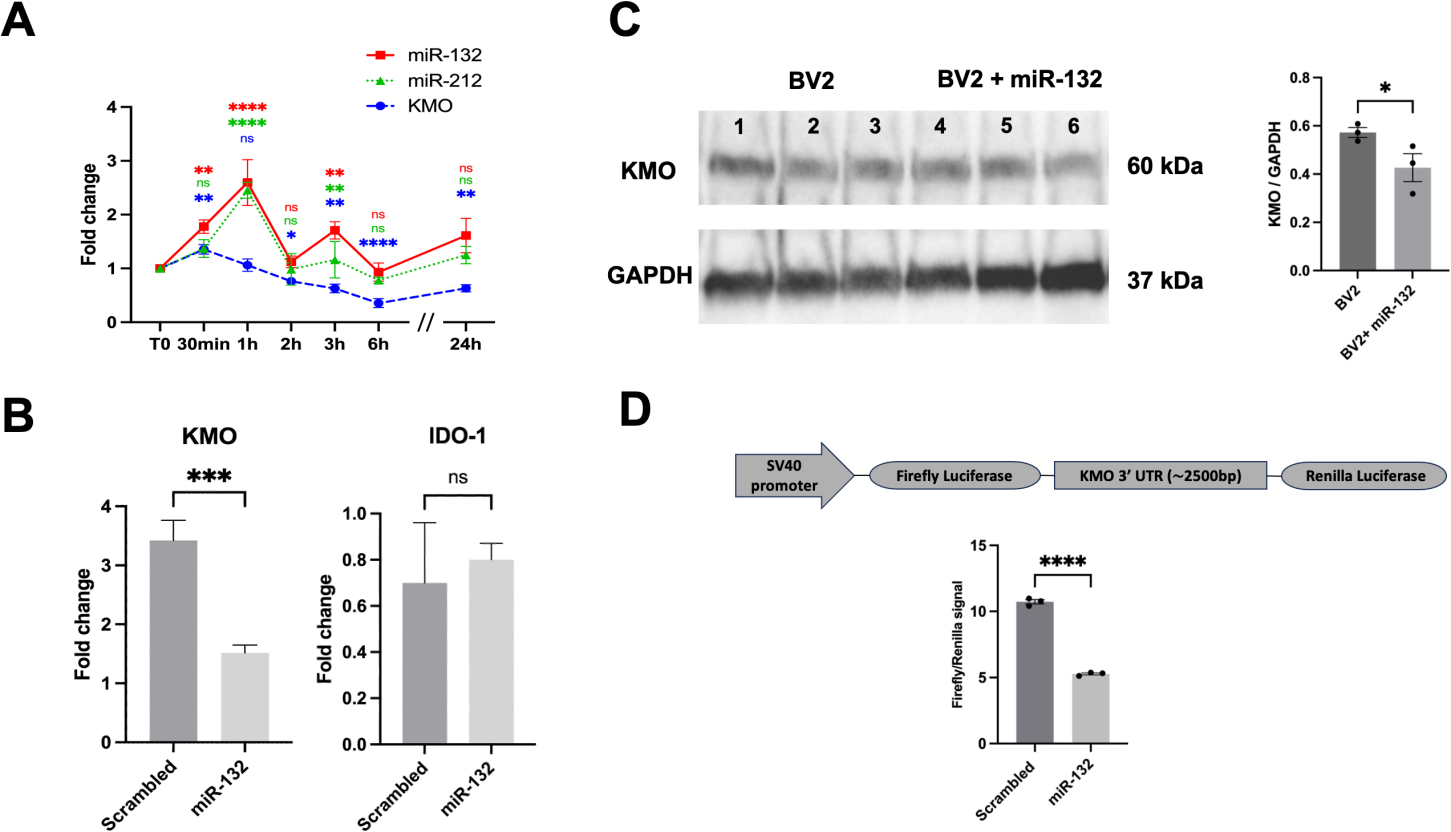
miR-132 modulates KMO enzyme activity in BV-2 microglia. **(A)** RT-qPCR results of KMO, miR-132, and miR-212 expression in BV-2 microglia cell culture after LPS treatment in different time points. The graph shows the mean values (n= 3 independent cultures) ± SEMs. The values were compared using one-way ANOVA test. Asterisks (*) indicate significant differences. *p < .05, **p < .01, and ***p < .001, ****p < .0001. **(B)** RT-qPCR results of KMO and IDO-1 gene expression in BV-2 microglia after transfection with miR-132 compared with that in the control (scrambled mimic). **(C)** Western blot and quantification of KMO protein levels after transfection with miR-132 compared to the control (scrambled mimic). Protein levels were normalized to GAPDH. **(D)** Schematic representation (not to scale) of the luciferase reporter construct used in this study (upper panel). SV40; Simian virus 40 promoter. HEK293 cells were transfected with miR-132 and a reporter construct containing the 3′UTR of murine KMO. Luciferase signal was normalized for transfection efficiency and graph represents the luciferase signals compared to the control condition (scrambled). All graphs show the mean values ​​(n= 3 independent cultures) ± SEMs. The values ​​were compared using the Student t test. Asterisks (*) indicate significant differences. *p < .05, **p < .01, and ***p < .001. ns, not significant.

The results obtained *in vivo* prompted us to investigate whether miR-132 or miR-212 could regulate KMO enzyme expression and QUIN production in isolated microglia. We first examined the effects of LPS treatment (100 ng/ml) on endogenous miR-132, miR-212, and KMO expression in BV-2 microglial cultures at various time points: 30 min, 1h, 2h, 3h, 6h, and 24h (**Figure 3A**). LPS exposure induced a rapid upregulation of miR-132, miR-212, and KMO expression. However, distinct temporal patterns were observed: KMO expression began to decline progressively after 30 min, whereas miR-132 and miR-212 levels peaked at 2 h post-treatment. These results are consistent with our earlier *in vivo* findings, suggesting that these miRNAs, particularly miR-132, which is more abundantly expressed, attenuate LPS-induced KMO expression in microglia.

To test this hypothesis, we transfected microglial cultures with miR-132 mimics to simulate its overexpression. RT-qPCR analysis revealed a significant downregulation of endogenous KMO mRNA levels in cells transfected with miR-132 compared to the control (scrambled mimic) (**Figure 3B**). This effect was not observed with the expression of another gene of interest in the KP pathway, IDO-1. We also observed a decrease in endogenous KMO protein levels under the same conditions (**Figure 3C** lanes 4-6). MiRNAs typically function by binding to conserved seed regions within the 3′UTR of their target mRNAs (38). However, a comprehensive search using current miRNA target prediction algorithms did not identify a conserved miR-132 or miR-212 binding site within the KMO 3′ UTR (data not shown). Interestingly, recent studies have highlighted the existence of alternative miRNA-binding mechanisms, such as seedless, imperfect, or non-canonical interactions, that can still mediate gene regulation (39–41).

To explore this possibility, we co-transfected HEK293 cells with a luciferase reporter vector containing the full-length KMO 3’UTR. We observed a significant reduction in luciferase (KMO) expression in the presence of miR-132 mimics compared to the negative control (scrambled) (**Figure 3D**), which is consistent with the mRNA and protein findings. Taken together, these results strongly suggest that miR-132 directly decreases KMO expression *via* its 3’UTR.

### miR-132 reduces QUIN metabolite production in BV-2 microglia

**Figure 4:**
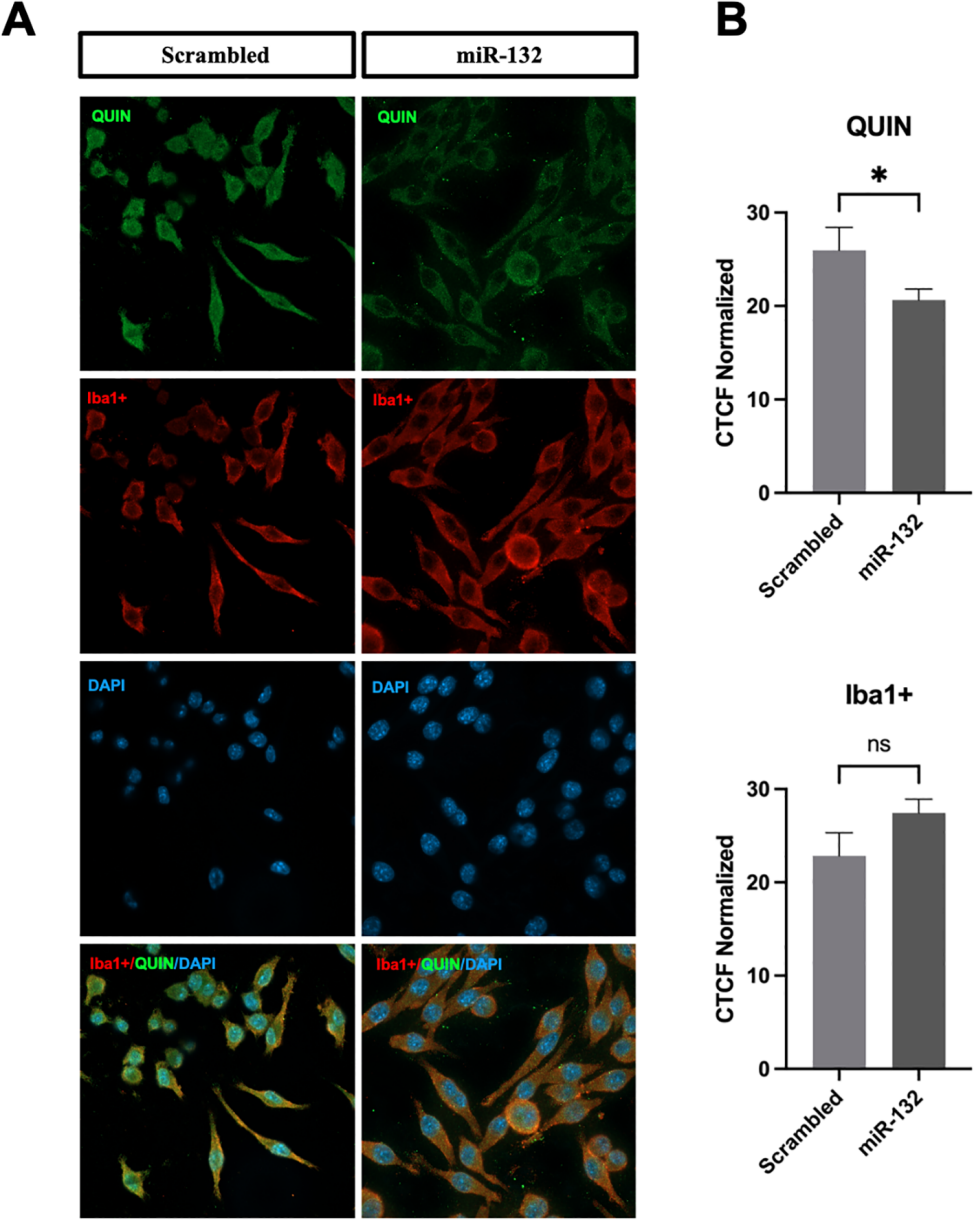
miR-132 reduces QUIN production in BV-2 microglia. **(A)** Immunofluorescence staining results of QUIN (in green) and BV-2 microglia (Iba1+ in red and DAPI in blue) transfected with control (scrambled) and miR-132. **(B)** Quantification of endogenous QUIN and Iba1+ staining of both conditions expressed in corrected total cell fluorescence (CTCF) normalized to the non-treated condition. All graphs show mean values (n = 15-30 cells per condition) ± SEMs. The values were compared using the Student t test. Asterisks (*) indicate significant differences. *p < .05. ns, not significant.

Finally, we investigated whether miR-132 modulates the production of QUIN metabolite in cultured microglia after immunofluorescent staining with specific anti-QUIN antibodies. Our results showed a significant reduction in QUIN levels (fluorescence signal) in BV-2 microglia transfected with miR-132 mimics compared to the control (**Figures 4A, B**). Notably, quantification of Iba1, a marker of microglial activity, showed no differences following miR-132 overexpression (**Figure 4B**).

## Discussion

In this study, we provide strong evidence that miR-132 regulates the expression and activity of KMO, the central enzyme of the kynurenine pathway (KP), following LPS-induced neuroinflammation. This is based on a combination of descriptive and functional data obtained *in vivo* in mice and validated using microglia cultures. Collectively, these results reveal a novel regulatory pathway by which miR-132 modulates KP metabolism, suggesting its therapeutic value in neuroinflammatory disorders.

The direct regulation of KMO by miR-132 was supported by luciferase reporter assays, which demonstrated a significant reduction in KMO 3′ UTR activity upon miR-132 overexpression. This result was further substantiated by the decreased KMO mRNA and protein levels observed in BV-2 microglial cells transfected with miR-132 mimics. To our knowledge, this is the first report to identify KMO as a direct target of miR-132, expanding the repertoire of miRNA-regulated enzymes in the kynurenine pathway. Prior studies have described the upstream regulatory effects of other miRNAs, such as miR-874-3p on IDO-1 (35) and miR-126-5p on TDO2 (34) in non-neuronal cells or tissues. This finding defines a novel epigenetic axis linking neuroinflammatory cues to metabolic outputs *via* miR-132-mediated repression of KMO. Interestingly, conventional miRNA target prediction tools (e.g., TargetScan) failed to identify a canonical seed match between miR-132/212 and the KMO 3 ′ UTR, suggesting a non-canonical binding mechanism. This highlights some limitations of *in silico* predictions and underscores the need for empirical validation in specific cellular contexts, particularly during neuroinflammation and possibly other stress cues. Our data align with the growing recognition that many biologically relevant miRNA-mRNA interactions occur through atypical or seedless binding sites, often involving partial complementarity or compensatory 3′ pairing. To dissect this mechanism further, future studies will require deletion mapping combined with 3 ′ UTR mutagenesis and possibly crosslinking assays to pinpoint the specific regions mediating miR-132 binding and repression.

MiR-132 is a well-documented modulator of neuronal homeostasis and neuroinflammation within the CNS. Beyond its known anti-inflammatory effects mediated by acetylcholinesterase inhibition (23, 24), miR-132 has also been shown to modulate microglial activation status (42). Our results provide new information on miR-132 mechanistic actions by demonstrating its direct involvement in the regulation of KP metabolism and QUIN accumulation. Additional potential mechanisms may further contribute to these modulatory effects. Indeed, miR-132 is known to negatively regulate NF-κB in different cell types (31, 43, 44), a transcription factor activated in inflammatory contexts that is correlated with higher KMO expression (45, 46). By targeting NF-κB, miR-132 can indirectly modulate the KP in other cell types, such as neurons or astrocytes, where miR-132 is also expressed, thereby altering neuron-microglia-astrocyte crosstalk through secreted factors or extracellular vesicles. Further studies are necessary to delineate the full spectrum of miR-132 targets and modulatory effects within the KP.

The early and exaggerated upregulation of pro-inflammatory cytokines (IL-12, TNF-α and IFN-γ) in miR-132/212 KO mice at 6 hours post-LPS administration further suggests that this miRNA cluster functions as a rapid negative feedback regulator during the acute phase of immune activation. Mechanistically, this may occur through miR-132 (and perhaps miR-212)-mediated targeting of upstream adaptor proteins such as TRAF6, TAB1, TAK1 and the STAT1 co-activator p300 (31, 47), thereby attenuating NF-κB and JAK/STAT signaling pathways, both central to cytokine gene transcription. The absence of this regulatory brake in knockout animals likely contributes to the amplified cytokine burst observed in early inflammation. By 24 hours post-injection, however, cytokine levels normalize in both WT and KO mice, likely due to the engagement of additional compensatory feedback mechanisms such as glucocorticoid signaling, anti-inflammatory cytokines, or SOCS protein induction. This dynamic highlights the temporal specificity of miR-132/212 action, acting primarily as an early-phase modulator of inflammatory signaling. Importantly, despite the resolution of the acute cytokine response, we observed sustained upregulation of IDO-1, KATII, and KMO expression in miR-132/212 KO mice at 24 hours post-LPS, a time point at which miR-132/212 levels had already returned to baseline in WT animals. This suggests that early loss of miR-132/212-mediated repression permits prolonged KP activation, with potential consequences for chronic neurotoxicity. The transient induction profile of miR-132/212 in response to LPS is consistent with prior studies (48), further supporting the notion that this cluster serves as a temporally restricted yet critical checkpoint in both inflammatory and metabolic regulation.

The sustained upregulation of KMO and prolonged QUIN accumulation after LPS treatment in miR-132/212 KO mice, suggest that miR-132 deletion (and perhaps in combination with miR-212) can create an extended neurotoxic metabolic state even after the resolution of inflammation. Interestingly, miR-132 is associated with the pathophysiology of neurodegenerative disorders, such as Alzheimer’s disease (AD), and is strongly associated with neuroinflammation. Decreased miR-132 (and miR-212) levels are constantly found in post-mortem AD brains, especially in cognitive function-related regions, such as the hippocampus and prefrontal cortex (27, 49). Downregulation is associated with synaptic impairment, hyperphosphorylation of the tau protein, and disrupted neuroplasticity characteristics in AD pathology (26, 50). Increased concentrations of QUIN have been identified in the brains of patients with AD (51), inducing excitotoxicity through overactivation of NMDA receptors and promoting oxidative stress. Lower levels of miR-132/212, as observed in AD patients, would therefore be linked to an increase in QUIN and the induction of its neurotoxic effects in these patients. Of course, the potential role of the miR-132-KMO-QUIN axis may function beyond AD and participate in other neurodegenerative or neuroinflammatory (ex: infection) cases.

Furthermore, during this study, we focused on microglia, the main source of QUIN in the brain and KMO is predominately expressed in these cells (51, 52). However, astrocytes also play a central role in kynurenine metabolism, particularly as the main source of KYNA. Indeed, future studies should include astrocyte-specific investigations to better understand the interplay between protective and neurotoxic branches of the KP and the potential regulatory role of miR-132/212 across different glial cell types.

Systemic LPS administration is well known to induce pronounced sickness behavior and cognitive impairments, largely driven by neuroinflammatory processes (53, 54). Although this relationship is well established, our study did not incorporate behavioral or functional assessments, primarily due to the moribund condition of several animals from both genotypes following LPS injection. This acute response limited the feasibility of conducting reliable behavioral analyses within our experimental timeframe. Future studies should systematically evaluate a range of behavioral domains to capture the full spectrum of neuroinflammatory and neurotoxic effects associated with miR-132/212 deficiency and QUIN accumulation. These assessments could include (1): general sickness behaviors, such as locomotor activity (open field test), body weight loss, food intake, and thermoregulation (2); motivational and affective-like behaviors, including anhedonia (sucrose preference test) and despair-related behaviors (forced swim test or tail suspension test); and (3) cognitive function and memory, assessed through novel object recognition (short-term memory), Y-maze (working memory), and Morris water maze (spatial learning and memory). We have already documented physiological, cognitive and motor aspects of miR-132/212 KO mice (36). Whether these become induced or exacerbated following LPS treatment remains an intriguing possibility.

Therapeutically, pharmacological KMO inhibition has been proposed as a strategy to shift KP metabolism toward neuroprotective kynurenic acid (KYNA) over neurotoxic QUIN. However, KMO inhibitors are hampered by poor brain penetration and low specificity (55). Our findings suggest that restoring miR-132 levels, particularly in microglia, may offer a biologically fine-tuned solution. Given the consistent downregulation of miR-132 in AD and the dual therapeutic impact of inhibiting inflammation and lowering QUIN toxicity, miR-132 mimics are clearly an attractive therapeutic compound for AD and associated disorders. Numerous strategies based on Cationic Polymer-Based nanoparticle carriers, such as poly (lactic acid-co-glycolic) acid (PLGA) (56) or exosomes, extracellular vesicles that have the natural capacity to cross the BBB (57–59), are now available to deliver miRNA mimics into the brain.

This study had some limitations. First, our analysis was limited to two acute time points (6 and 24 hours) following LPS treatment. While this design enabled us to capture the early regulatory role of miR-132/212 on inflammatory cytokines and KP enzymes, it does not capture the longer-term dynamics of KP regulation or potential compensatory mechanisms that may emerge over time. Future investigations will therefore require chronic time points (e.g., 3, 7 and 14 days post-injection) to assess the persistence or resolution of KMO upregulation and QUIN accumulation in miR-132/212 KO mice. These extended studies will also be critical to determine whether the early molecular changes observed in the acute phase lead to brain cell remodeling or delayed behavioral consequences. In addition, the LPS model used in this study may not fully replicate the chronic inflammatory state associated with neuroinflammatory or neurodegenerative diseases. Furthermore, while we attributed QUIN production to microglia, mRNA quantifications and KP metabolomic analyses were conducted on whole-brain tissue; cell-type-specific profiling would provide greater resolution. Future approaches could incorporate fluorescence-activated cell sorting (FACS), single-cell or spatial transcriptomics, and conditional knockout models to dissect the relative contributions of individual cell types, including astrocytes. This will be essential to determine whether the regulatory effect of miR-132 on KMO is microglia-specific, or part of a broader intercellular regulatory network within the CNS during neuroinflammation.

Furthermore, although widely adopted, BV-2 microglia cells differ from primary microglia in terms of responsiveness and transcriptomic profiles. Further experiments using primary microglia and/or IPSC microglia-like cells are necessary to validate these results. Finally, our study relies exclusively on murine models, both *in vivo* and *in vitro*. While these models are well-established, they do not fully capture the complexity or species-specific regulation present in the human brain. To strengthen translational relevance, future studies will need to incorporate human iPSC-derived microglia and 3D brain organoids to validate the regulatory relationship between miR-132 and KMO in a human genetic and cellular context. These systems will allow us to assess cell-specific responses under inflammatory conditions and test therapeutic delivery of miR-132 mimics in human-derived tissue-like environments. This approach will help bridge the gap between experimental models and human neuroinflammatory disorders.

In summary, we identified miR-132 as a potent regulator of KMO in microglia, which plays a key role in controlling QUIN production during neuroinflammation. This suggests a novel epigenetic mechanism for the regulation of KP activity. Interestingly, the loss of miR-132, as observed in AD, could contribute to prolonged neurotoxic metabolism and impaired microglial function. Restoring miR-132 brain levels may offer a promising way to reduce neuroinflammation and protect against QUIN-driven excitotoxicity in neurodegenerative diseases.

## Data Availability

The datasets presented in this study can be found in online repositories. The names of the repository/repositories and accession number(s) can be found below: PXD066936 (ProteomeXchange).
